# Women at disadvantage – sex differences in time between dialysis initiation and kidney transplant evaluation or listing: a single-center experience

**DOI:** 10.1186/s12882-025-04663-6

**Published:** 2025-12-01

**Authors:** Lena-Sophia Wandinger, Lara Ploeger, Julia Menne, Birgit Kortus-Goetze, Joachim Hoyer, Martin Russwurm, Johannes Wild

**Affiliations:** 1 Department of Internal Medicine and Nephrology, Marburg University, University Hospital Marburg, 35043 Marburg, Germany; 2 Institute of Pharmacology, Marburg University, Marburg, Germany

**Keywords:** Barriers for transplantation, Gender inequalities, Kidney transplantation, Transplant evaluation process

## Abstract

**Background:**

Kidney transplantation remains the most effective treatment for end-stage kidney disease, but sociodemographic differences, including sex and gender differences, remain a significant challenge. There is evidence that women receive fewer transplants than men, but whether gender influences the timing of transplant evaluation and waitlisting has not been analyzed yet. Therefore, we aimed to investigate gender differences in the time intervals between dialysis initiation, transplant evaluation and waitlisting.

**Methods:**

We retrospectively analyzed 182 patients listed for kidney transplantation in the last quarter of 2023 (Key date 1st October 2023) or positively evaluated for listing for kidney transplantation from 2017 to 2023 at the University Medical Center Marburg, Germany. Besides baseline characteristics, we collected data on dialysis initiation, start of transplant evaluation and time from evaluation to waitlisting (Ethics approval Nr. 24-97RS, Philipps University Marburg, Germany).

**Results:**

In our cohort, we did not find significant overall gender differences in the time intervals from dialysis initiation to transplant evaluation or listing. However, male patients undergoing peritoneal dialysis or scheduled for living donor transplants had significantly shorter evaluation and waitlisting times compared to their female counterparts.

**Conclusions:**

The findings from our single-center analysis with a limited sample size suggest that gender disparities are pronounced in specific subgroups, with men generally benefiting from faster access to the waitlist. The results highlight the importance of recognizing gender disparities in transplantation processes to ensure equitable access for all patients. Future research should focus on interventions that address these inequalities and promote gender-sensitive nephrology care.

**Supplementary Information:**

The online version contains supplementary material available at 10.1186/s12882-025-04663-6.

## Background

Kidney transplantation is the most effective therapeutic option for patients with end-stage kidney disease (ESKD) [1]. However, the global shortage of organs remains a significant and well-documented challenge [2]. Beyond the scarcity of organs, the transplant availability is further hindered by a range of socio-demographic barriers, such as race, median income, and residential location [3]. These disparities have been thoroughly studied and continue to be a key focus of research aimed at raising awareness and improving access to transplantation for all patients [4]. Additionally, sex and gender are important predictors of whether a patient successfully makes it onto the transplant waitlist. Sex and gender are often used completely synonymously but are increasingly understood and respected as two different entities. Sex is the biological attribute, while gender encompasses the social, cultural and psychological aspects of a person [5]. For more than 25 years, research has consistently shown that women, or persons who identify as such, receive significantly fewer kidney transplants than men - a disturbing disparity that has been observed and replicated across multiple cohorts and continents [6–8]. In eight European countries including Austria, Belgium, Croatia, Germany, Hungary, Luxembourg, the Netherlands, and Slovenia, Eurotransplant, a non-profit organization, coordinates the allocation of donor organs [9]. In general, the process towards transplantation begins when the nephrologist refers the patient to a transplantation center. The timing of this referral represents a crucial opportunity to mitigate delays. Ideally, patients with pre-terminal kidney disease should be informed about the potential for a kidney transplantation and considered for living donor options before the need for dialysis arises. In a first step to waitlisting, aiming for the shortest possible duration from the initiation of dialysis to being placed on the transplant list is crucial for improving outcomes [10]. The second stage of the process occurs upon referral, when the patient is evaluated at the transplant center to ascertain their suitability for transplantation. This stage is concluded with either listing or rejection of the patient. In Germany, this decision is made based on a comprehensive assessment according to the admission criteria outlined in the organ transplantation guidelines, as per German transplant law [11]. To avoid any delays, it would be essential to complete all required examinations as promptly as possible.

Studies have documented that women receive kidney transplants less frequently than men [12], the same holds true for liver and hearts transplants [13]. However, to our knowledge, no study has yet examined whether sex or gender differences exist in the time required to complete the different steps leading to kidney transplantation. In a retrospective, single-center analysis, we sought to investigate whether female sex or gender serves as a significant predictor for the duration of the two critical time intervals leading to kidney transplant listing. Specifically, we aimed to determine if sex or gender influences both the time elapsed between the initiation of dialysis and referral to a transplant center, as well as the length of the subsequent evaluation process culminating in either inclusion on the transplant waitlist or exclusion.

## Methods

We conducted a retrospective analysis including all patients listed for kidney transplantation at the University Medical Center Marburg, Germany in the last quarter of 2023 (Key date 1st October 2023) or positively evaluated for listing for kidney transplantation from 2017 to 2023 at the Transplant Center of the University Medical Center Marburg (*N* = 296). The study was approved by the institutional ethics review board (24-97RS, Ethics Committee of the Philipps University Marburg, Germany). All procedures were conducted in accordance with the declaration of Helsinki. We included only kidney transplant–naïve patients, as all of them underwent standardized inpatient transplant evaluation at our center. In contrast, patients evaluated for a second or third transplant are managed on a highly individualized basis and were therefore considered too heterogeneous for inclusion in our retrospective analysis.

Data were obtained from the digital patient records collected as part of the standardized transplant evaluation process at our center. The evaluation procedure at our transplant center is highly standardized. Initially, patients are referred by an external dialysis center. For this referral, a recent medical report is required, including information on the underlying kidney disease, the clinical course, the initiation of dialysis, and the dialysis modality. Subsequently, patients undergo a standardized assessment protocol, which is primarily conducted in an inpatient setting. This protocol strictly follows the recommendations of the *KDIGO Clinical Practice Guideline on the Evaluation and Management of Candidates for Kidney Transplantation* [14]. Once all required examinations have been completed, the case is discussed in the interdisciplinary transplant conference, and—if the consensus is positive—the patient is listed with Eurotransplant. For the present analysis, we extracted all available data regarding the initiation of dialysis, cause of ESKD, dialysis modality, and timing of the transplant evaluation process, including the time interval between the first day of evaluation and listing. In addition, we analyzed the number of patients evaluated and listed for living donor kidney transplantation (LDKT). Gender and sex information was derived from patient records. Biological sex was confirmed using physical examination findings and abdominal computed tomography imaging, and self-identified gender was verified based on the recorded name.

Statistical analyses were conducted using GraphPad Prism software (version 10; GraphPad Software Inc., San Diego, CA, USA). Data are presented either as individual values or as the mean ± standard error of the mean (S.E.M). Normality of the data was assessed using the D’Agostino & Pearson test, and outliers were identified using the ROUT method with a Q value of 1% (maximum desired False Discovery Rate). Descriptive statistics for comparisons of key patient characteristics between female and male patients evaluated for kidney transplantation are reported as medians with standard deviations (SD) or as absolute numbers with corresponding percentages. For continuous variable comparisons, we applied the Mann-Whitney U test or t-test, depending on the distribution, while categorical variables were assessed using Fisher’s exact test. Additionally, Kruskall-Wallis Test with Dunn’s multiple comparison test, Welch and Brown-Forsythe ANOVA and simple linear regression were employed where applicable. Statistical significance was defined as a p-value of < 0.05, with significance levels indicated as follows: *p < 0.05, **p < 0.01, ***p < 0.001.

## Results

Overall, 296 patients were included in the waiting list. Children (*N* = 31), patients with a change of transplant center (*N* = 33) or incomplete data sets (*N* = 50) had to be excluded. A total of 182 complete datasets remained for analysis. Regarding baseline characteristics, in our cohort of 182 patients evaluated and listed for kidney transplantation (Table 1), median age at kidney transplant evaluation was significantly lower for female patients (45.84 +/- 12.37 years) compared to male patients (50.85 +/- 11.68 years).

**Table 1:** Baseline characteristics of transplant evaluation patients stratified by gender. t-Test (Age) or Fisher‘s exact test (all other parameters)

	All	Female	Male	*p*-value
	*n* = 182	*n* = 64(35.2%)	*n* = 118(64.8%)	
**Age at transplant evaluation**, median (SD)	49.09 (12.4)	45.84 (12.9)	50.85 (11.7)	**0.009**
**Form of Renal Replacement Therapy**				
Hemodialysis (%)	136 (74.7)	46 (71.98)	90 (76.3)	0.59
Peritoneal Dialysis (%)	35 (19.2)	15 (23.4)	20 (16.9)	0.32
Preemptive transplant (%)	11 (6.04)	3 (4.6)	8 (6.8)	0.75
**Form of transplant**				
Living donor (%)	35 (19.3)	14 (21.9)	21 (17.8)	0.55
Deceased donor (%)	147 (80.8)	50 (78.1)	97 (82.2)	0.55
**Cause of ESKD**				
Diabetes (%)	42 (23.0)	12 (18.8)	30 (25.4)	0.36
IgA Nephritis (%)	25 (13.7)	7 (10.9)	18 (15.2)	0.50
Hypertension (%)	24 (13.2)	6 (9.4)	18 (15.2)	0.36
Cystic Kidney disease (%)	24 (13.2)	11 (17.2)	13 (11.0)	0.25
FSGS (%)	7 (3.8)	2 (3.1)	5 (4.2)	0.99
Congenital anomalies (%)	6 (3.3)	5 (7.8)	1 (0.9)	0.02
Granulomatosis with polyangiitis (%)	6 (3.3)	3 (4.7)	3 (2.5)	0.43
Tubulointerstitial Nephritis (%)	6 (3.3)	2 (3.1)	4 (3.4)	0.99
Alport Syndrome (%)	4 (2.2)	0	4 (3.4)	0.29
Membranous GN (%)	3 (1.6)	0	3 (2.5)	0.55
Other/Unknown (%)	35 (19.2)	16 (25.0)	19 (16.1)	0.17

In our cohort, we could use the terms sex and gender synonymously, as all biologically female persons also identified as women, and the same applied to included male patients. The majority of patients (74%) undergoing kidney transplant evaluation were treated with hemodialysis (HD). The percentage of female patients on HD was 72%, while for male patients it was slightly higher at 76%. However, this difference was not statistically significant (p-value = 0.59), indicating similar rates of HD use between genders. Percentages of peritoneal dialysis (PD) did also show no significant difference between both genders. Among patients evaluated for kidney transplantation, the majority (81%) were considered for deceased donor kidney transplantation (DDKT), while 19% were evaluated for living donor kidney transplantation (LDKT). There was no significant difference in the rates of evaluations for deceased versus living donor transplants between male and female patients. In our single-center cohort, no significant differences in underlying kidney disease were observed between the two genders.

**Fig. 1:**
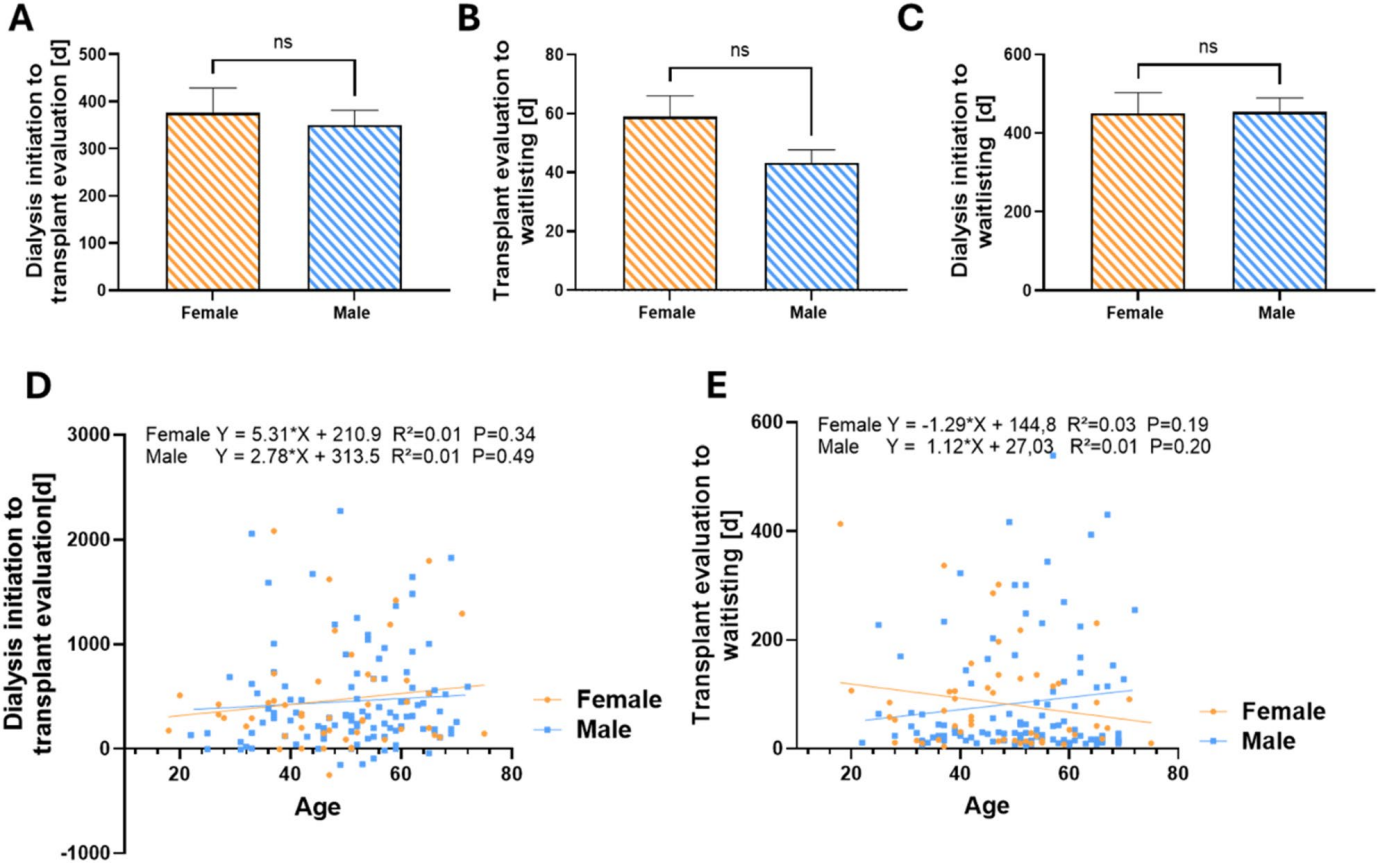
Respective time intervals as a function of recipient’s gender. Time span from dialysis initiation to transplant evaluation (**A**), transplant evaluation to waitlisting (**B**) and dialysis initiation to waitlisting (**C**) in female and male patients. Mann-Whitney test (*n* = 46/97). (**D**) Simple linear regression between age in years and time from dialysis initiation to transplant evaluation in days. (**E**) Simple linear regression between age in years and time from transplant evaluation to waitlisting in days. (ns = not significant)

We analyzed the time interval between dialysis initiation and listing, which was further dichotomized, firstly, in the interval between dialysis initiation and the start of transplant evaluation, and secondly the interval between the start of evaluation and definitive listing for transplantation. In our cohort, the time from dialysis initiation to transplant evaluation was 375.7 ± 349.8 days for women, compared to a slightly shorter interval of 359.7 ± 309.2 days for men (Fig. 1A). However, this difference was not statistically significant. The same trend appeared for the interval between the start of evaluation and listing. Here, the duration was 58.9 ± 43.2 days for female patients, compared to 53.8 ± 43.6 days for male patients (Fig. 1B).

Overall, no significant differences were observed between men and women in the interval between start of dialysis and listing for transplantation across our overall cohort (Fig. 1C). Recipient age did also not influence time between dialysis initiation and kidney transplant evaluation (Fig. 1D) or transplant evaluation to listing (Fig. 1E) in the overall cohort, again also without any differences between both genders. An analysis of the three examined intervals, stratified by the year of transplant evaluation between 2018 and 2023, revealed no significant differences in temporal trends—neither between females and males nor over time (Supplemental Fig. 1). In the small cohort analyzed, no impact of the COVID-19 pandemic on the time to transplant listing could therefore be demonstrated.

**Fig. 2:**
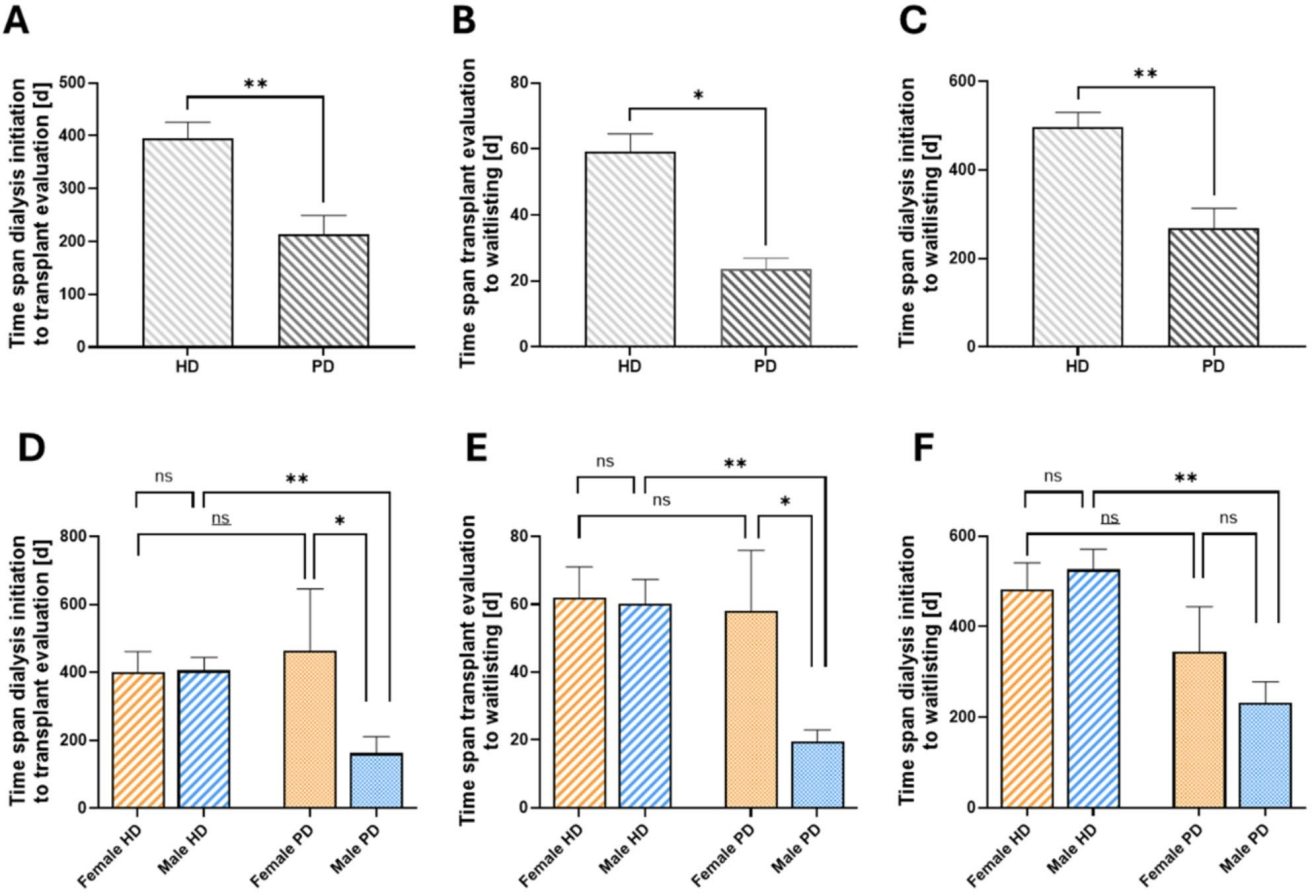
Respective time intervals as a function of RRT modality. Time span from dialysis initiation to transplant evaluation (**A**), transplant evaluation to waitlisting (**B**) and dialysis initiation to waitlisting (**C**) in patients treated with hemodialysis (HD) or peritoneal dialysis (PD). Mann-Whitney test (*n* = 119/34). Gender-stratified analysis for time span from dialysis initiation to transplant evaluation (**D**), transplant evaluation to waitlisting (**E**) and dialysis initiation to waitlisting (**F**) in HD or PD patients. Kruskal-Wallis test with Dunn’s test for multiple comparisons; *n* = 38 female HD/82 male HD/8 female PD/16 male PD. (ns = not significant)

In the next step, patients were stratified according to their dialysis modality. Here, we found that patients undergoing HD had significantly longer intervals until evaluation and listing for transplantation (Fig. 2A-C). These differences were statistically significant across all time intervals examined. Both the interval from dialysis initiation to the start of evaluation (394.4 +/- 213.5 days for HD vs. 342.6 +/- 207.9 days for PD) and from the start of evaluation to listing (59.0 +/- 58.6 days for HD vs. 23.6 +/- 14.4 days for PD) were significantly shorter for PD patients. The total time from dialysis initiation to listing also showed a significant difference (496.4 +/- 357.2 days for HD vs. 267.1 +/- 212.9 days for PD).

In a gender-separated analysis, the significantly shorter time intervals only were observed in male patients: time from dialysis initiation to transplant evaluation (405.2 +/- 346.4 days for HD vs. 161.5 +/- 195.9 days for PD, *p* = 0.002) and from evaluation to listing (60.7 +/- 63.4 days for HD vs. 19.6 +/- 11.8 days for PD, *p* = 0.01) were significantly shorter in males, while in women the time differences between PD and HD were not statistically significant (Fig. 2D-E).

**Fig. 3:**
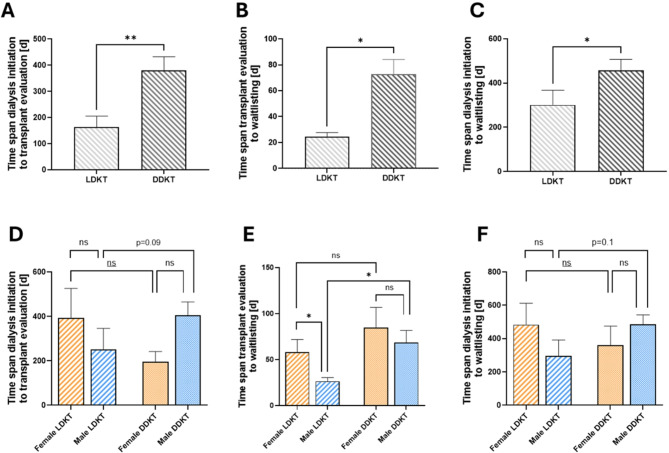
Respective time intervals as a function of donor modality. Time span from dialysis initiation to transplant evaluation (**A**), transplant evaluation to waitlisting (**B**) and dialysis initiation to waitlisting (**C**) in living donor kidney transplant (LDKT) or deceased donor kidney transplant (DDKT) recipients. Mann-Whitney test (*n* = 25/44). Gender-stratified analysis for time span from dialysis initiation to transplant evaluation (**D**), transplant evaluation to waitlisting (**E**) and dialysis initiation to waitlisting (**F**) in LDKT and DDKT recipients. Kruskal-Wallis test with Dunn’s test for multiple comparisons (**D/F**) or Welch and Brown-Forsythe ANOVA (**E**) (*n* = 12 female LDKT, 13 male LDKT, 9 female DDKT, 34 male DDKT). (ns = not significant)

Finally, we examined whether the time intervals from dialysis initiation to transplant evaluation and listing differed between patients receiving living donor and deceased donor transplantations. Patients evaluated for planned LDKT were referred significantly earlier than DDKT patients, (164.4 +/- 210.3 days from dialysis initiation to transplant evaluation for LDKT vs. 379.7 +/- 348.4 days for DDKT, p = 0.006), (Fig. 3A)

Additionally, the group of LDKT recipients completed the evaluation process significantly faster (24.6 +/- 15.6 days for LDKT vs. 72.9 +/- 74.9 days for DDKT, *p* = 0.02), leading to a significantly shorter time until they were listed as eligible for transplantation (Fig. 3B-C). However, this was not consistent across both genders. Once again, it was the female patients in our cohort who did not show significant differences between LDKT and DDKT (Fig. 3D-F). Males evaluated for LDKT not only had shorter time intervals from dialysis initiation to transplant evaluation (250.9 +/- 343.0 days for LDKT vs. 404.8+/- 351.7 days for DDKT, *p* = 0.09) but also completed the evaluation process significantly faster (26.4 +/- 17.2 days for LDKT vs. 68.7+/- 75.4 days for DDKT, *p* = 0.02) than male patients evaluated for DDKT.

## Discussion

The aim of our investigation was to investigate potential sex or gender disparities in the time intervals leading to kidney transplantation listing. In our retrospective analysis of a single-center cohort, no significant overall differences regarding the time from dialysis initiation to transplant listing were observed between male and female patients in the overall cohort. However, we identified pronounced disparities within the PD and the LDKT patient subgroups. Female patients treated with PD or scheduled for a LDKT had significantly longer time intervals from dialysis initiation to evaluation, as well as from evaluation to listing for transplantation in both subgroups. In contrast, no significant differences were observed in listing intervals between women according to RRT or donor modality. As a result, the overall time to listing was notably longer in women treated with PD or evaluated for a LDKT, pointing at ongoing differences in the transplantation process between male and female patients. Gender disparities are well-documented across various fields of medicine, particularly in cardiovascular disease, where substantial evidence shows that women often experience delayed diagnosis and undertreatment [15–17]. Awareness is also growing in nephrology regarding the gender disparities in prevalence and care of patients with kidney disease, particularly highlighting significant differences between females and males with chronic kidney disease and kidney transplantation [18, 19]. There are various analyses demonstrating that men are significantly more frequently listed for [20] and undergoing kidney transplantation [21, 22]. These data are consistently reported in different cohorts [23, 24] underscoring the significantly reduced access to the transplant waitlist for female patients, even after adjusting for age and key comorbidities [8]. However, it should also be noted that other authors challenge this clear disadvantage for women, citing varying prevalences of chronic kidney disease and the complex factors influencing these disparities that might extend beyond biological sex [13].

Our data fit well in the body of literature regarding the overall gender distribution of patients enlisted for kidney transplantation. In our cohort, 35.2% of the patients examined were female. This finding is corroborated by registry data, which indicates that 39% of individuals on the United Network for Organ Sharing and Eurotransplant kidney transplant waitlists are female, and 37% of those on the kidney transplant waitlist across German transplant centers are female. Several factors are suspected to contribute to the reduced access to transplantation for women compared to men: This not only begins with a reduced likelihood of discussing transplantation as a treatment option [25]. Also medical factors, such as the presence of panel-reactive antibodies or autoimmune diseases are known drivers of sex-inequalities in transplantation access for women: Obviously, pregnancy only occurs in women and is a known factor of alloimmunization [26]. These sex-specific disparancies have been shown to contribute to inequality in access to transplantation in women [27]. Although studies investigating this impact of preganancy induced PRA and access to kidney transplant are lacking, the unfavorable effect of alloimmunization through pregnancy can surely be deemed self-evident for potential women kidney transplant recipients. Autoimmune diseases are much more common in women [28]: Although there is no concrete evidence, it seems conceivable that active autoimmune disease might somehow be considered an obstacle to the initiation of transplant evaluation or transplantation itself, whether implicitly or explicitly.

While previous studies have examined reasons for the underrepresentation of women on transplant waitlists, our research focused on whether sex differences cause longer time intervals from the initiation of dialysis to evaluation and listing for transplantation. To our best knowledge, no study has yet investigated gender differences in the time from the start of dialysis to inclusion on the transplant waitlist, so we cannot compare our data with the existing literature. Without considering gender differences, the median duration of approximately one year from dialysis initiation to listing for transplantation in our cohort is comparable to German registry data [29]. It is noteworthy that in our cohort, the time elapsed between the commencement of the evaluation process and the final registration on the waiting list was approximately two months. This evaluation time is therefore much shorter than that reported from centers in other European countries. A report from Belgium cites an average evaluation time of 8.6 months [30], while a study from a French kidney transplant center reports a duration of 8.1 months for patients to complete the evaluation process [31]. In the US 23.4% of patients fail to complete the evaluation process within one year [32]. We explain our shorter time frames for patients of both genders by the fact that the standard operating procedure at our center provides for a strict assessment in an inpatient setting. As a result, all necessary examinations are carried out within a few days and a decision on listing is possible by interdisciplinary consensus directly after the inpatient assessment.

In our small single-center cohort, we did not observe any temporal differences across the years of transplant evaluation with respect to the time to evaluation or waitlisting. Although the impact of the COVID-19 pandemic on organ donation and transplantation rates has been well established [33, 34], our findings suggest that, within this cohort, the pandemic did not substantially affect the time from dialysis initiation to transplant evaluation or waitlisting. These results should be interpreted with caution, as the limited sample size and single-center design restrict the generalizability of our findings. Larger, multicenter studies are needed to confirm these observations and to better understand potential pandemic-related influences on time spans to transplant evaluation or waitlisting.

Over 20 years ago, Snyder et al. provided data from the US pointing out that PD patients are 50% more likely to be evaluated for kidney transplantation compared to HD patients. Similar results were subsequently published for the UK [35] and another US cohort [36]. The reason why PD patients are more likely to be listed and therefore to receive a kidney transplantation compared to HD patients is still unclear. It seems probable that this advantage is mediated by the fact that PD patients tend to be younger and to have fewer comorbidities than HD patients. Further, it is conceivable that among patients and their physicians PD is the preferred dialysis modality for transplantation candidates, seen more as a bridge than a destination therapy. While ethnicity, age, insurance status and socioeconomic status are fairly well-known and frequently discussed predictors of progress to the waitlist [37], dialysis modality as predictor of listing has not received much attention. While there is convincing data that PD patients are transplanted more frequently, there was no evidence that the time span from dialysis initiation to entering the waitlist is shorter for them. In the cohort we describe, there were significant differences between dialysis modalities as regard to listing time. PD patients as a whole showed shorter turnaround times in all three dimensions than HD patients. One possible explanation for this difference may lie in the fundamental differences between the two RRT modalities in terms of initiation urgency: PD is typically initiated in patients whose need for dialysis is not acutely urgent. These patients generally undergo a one- to two-week training period, which may provide additional opportunities to discuss kidney transplantation as a long-term treatment option or to initiate transplant evaluation. In contrast, HD is more often started under urgent circumstances, leaving less time for pre-dialysis counseling. However, during chronic treatment, HD patients have more frequent contact with healthcare providers compared to those on PD. In our combined analysis of both genders, patients who were treated with PD were significantly faster on the way to listing than patients who received HD. Most interestingly, a gender-stratified analysis then revealed that in our cohort, this advantage exists exclusively for male patients. The reasons for this remain speculative: It is conceivable that factual access to transplantation is restrained especially for women on PD because of fixed gender roles (e.g. household responsibilities, childcare and caring for the elderly). Nevertheless, the data we have collected cannot provide any causation or explanation, but it can serve as a firm reminder that female PD patients should be seen as potential transplantation candidates and thus, evaluated in a timely fashion.

In our opinion, one of the most remarkable and alarming results of our work represents the sex difference in time from dialysis initiation to waitlisting in LDKT. It is known that women are significantly overrepresented among living kidney donors [38, 39]. Data show that 60% of all living kidney donors are women. Again, there is no definitive data available regarding the cause of these discrepancies However, it is hypothesized that the higher propensity for female individuals to engage in charitably giving may be influenced by sociocultural factors [5, 40]. Rota-Musoll et al. have shown that women who are biologically related to the recipient see their donation as a natural and logical thing to do [41]. Interestingly, married women donate for gender role reasons, such as indirect protection of their children, but also as a form of self-empowerment and as a personal benefit [41]. In addition to these published data regarding gender differences in living kidney donation willingness, our data now suggests that there are also differences on the way to waitlisting. Of course, it is tempting to assume similar causes as discussed above. However, as we did not ask about such reasons, our data are not suitable to provide an answer.

Our study is subject to several important limitations. It reflects the experience of a single center, which limits the generalizability of the findings to other institutions. Since transplant centers are known to differ greatly in their processes and procedures regarding transplant candidacy evaluation and waitlisting practices [42], this is an essential limitation that must be clearly stated. Consequently, the applicability of our results to broader clinical practice remains uncertain. Furthermore, due to the relatively small sample size, the conclusions drawn are inherently exploratory and should be regarded as hypothesis-generating rather than definitive. Age at CKD diagnosis could not be reliably determined in our cohort, limiting the assessment of potential sex- or gender-related differences in both the timing of diagnosis and the interval from CKD diagnosis to transplant evaluation. Finally, since all patients in our study self-identified in a cis-gender manner, meaning that all biological females identified as women (and males as men, respectively) we cannot draw any conclusion regarding particularly trans-gender inequalities from our data.

Despite these limitations, we consider our findings to be an important contribution to gender-sensitive medicine in nephrology, particularly in the field of transplant nephrology. A strength and important implication of our study is that stratifying patients by gender can reveal contradictory effects: while PD is a positive predictor of faster transplant evaluation turnaround time in the overall cohort and in men, this effect is not observed in women. Moreover, while several studies have addressed gender or sex-specific differences in kidney transplantation frequency, to our knowledge, we can provide first data examining the impact of gender differences on the time spans from dialysis initiation to transplant evaluation and listing.

Gender disparities in medical care remain a significant challenge in contemporary practice. Addressing this issue requires, as a first step, recognition and acknowledgment of its existence. Furthermore, clear communication, in our view, represents the most important component in reducing the detected inequalities. The substantial benefits of timely transplantation must be explicitly conveyed by every nephrologist. Every patient, regardless of gender, must be fully informed, and individual barriers on the path to transplantation must be addressed. In particular, concerns about providing for one’s family, in our view, often reflect a misperception, as transplantation improves both life expectancy and quality of life [43]. It therefore remains a central responsibility of medical practice to recognize existing gender disparities and actively address them. Only through this approach, early interventions can be implemented to ensure that female patients achieve access to transplantation at the same pace as male patients.

## Supplementary Information

Below is the link to the electronic supplementary material.

Supplementary Material 1

## Data Availability

All data are presented in the manuscript and its accompanying files.

## Abbreviations

CKD: Chronic Kidney Disease
DDKT: Deceased Donor Kidney Transplant
ESKD: End-Stage Kidney Disease
FSGS: Focal Segmental Glomerulosclerosis
GN: Glomerulonephritis
HD: Hemodialysis
KDIGO: Kidney Disease: Improving Global Outcomes
LDKT: Living Donor Kidney Transplant
PRA: Panel Reactive Antibodies
PD: Peritoneal Dialysis
RRT: Renal Replacement Therapy

## References

[1] Tonelli M, et al. Systematic review: kidney transplantation compared with Dialysis in clinically relevant outcomes. Am J Transpl. 2011;11(10):2093–109. 10.1111/j.1600-6143.2011.03686.x 21883901

[2] Lewis A, et al. Organ donation in the US and europe: the supply vs demand imbalance. Transpl Rev (Orlando). 2021;35(2):100585. 10.1016/j.trre.2020.100585 33071161

[3] Alexander GC, Sehgal AR. Barriers to cadaveric renal transplantation among blacks, women, and the poor. JAMA. 1998;280(13):1148–52. 9777814 10.1001/jama.280.13.1148

[4] Zhang C, Mathur AK. Breaking barriers and bridging gaps: advancing Diversity, Equity, and inclusion in kidney transplant care for black and Hispanic patients in the united States. Transpl Int. 2023;36:11455. 37829616 10.3389/ti.2023.11455 PMC10565005

[5] Katz-Greenberg G, Shah S. Sex and gender differences in kidney transplantation. Semin Nephrol. 2022;42(2):219–29. 35718368 10.1016/j.semnephrol.2022.04.011 PMC10065984

[6] Schaubel DE, et al. Sex inequality in kidney transplantation rates. Arch Intern Med. 2000;160(15):2349–54. 10927733 10.1001/archinte.160.15.2349

[7] Purdy A, et al. Sex differences in renal transplantation in Canada. J Womens Health Gend Based Med. 1999;8(5):631–5. 10839649 10.1089/jwh.1.1999.8.631

[8] Melk A, et al. Sex disparities in Dialysis initiation, access to waitlist, transplantation and transplant outcome in German patients with renal disease-A population based analysis. PLoS ONE. 2020;15(11):e0241556. 33180815 10.1371/journal.pone.0241556 PMC7660568

[9] Persijn GG. Allocation of organs, particularly kidneys, within Eurotransplant. Hum Immunol. 2006;67(6):419–23. 16728263 10.1016/j.humimm.2006.03.008

[10] Naylor KL, et al. Pre-transplant maintenance Dialysis duration and outcomes after kidney transplantation: A multicenter population-based cohort study. Clin Transpl. 2022;36(3):e14553. 10.1111/ctr.14553 34897824

[11] Zecher D, et al. Regional differences in waiting times for kidney transplantation in Germany. Dtsch Arztebl Int. 2023;120(23):393–9. 37097064 10.3238/arztebl.m2023.0098 PMC10433364

[12] Ahearn P, et al. Sex disparity in Deceased-Donor kidney transplant access by cause of kidney disease. Clin J Am Soc Nephrol. 2021;16(2):241–50. 33500250 10.2215/CJN.09140620 PMC7863650

[13] Melk A, et al. Equally interchangeable? How sex and gender affect transplantation. Transplantation. 2019;103(6):1094–110. 30747857 10.1097/TP.0000000000002655

[14] Chadban SJ, et al. KDIGO clinical practice guideline on the evaluation and management of candidates for kidney transplantation. Transplantation. 2020;104(4S1 Suppl 1):S11–103. 32301874 10.1097/TP.0000000000003136

[15] Crea F, Battipaglia I, Andreotti F. Sex differences in mechanisms, presentation and management of ischaemic heart disease. Atherosclerosis. 2015;241(1):157–68. 25988360 10.1016/j.atherosclerosis.2015.04.802

[16] Ricci B, et al. Atypical chest pain in ACS: A trap especially for women. Curr Pharm Des. 2016;22(25):3877–84. 26956231 10.2174/1381612822666160309115125

[17] Vaccarino V, et al. Presentation, management, and outcomes of ischaemic heart disease in women. Nat Rev Cardiol. 2013;10(9):508–18. 23817188 10.1038/nrcardio.2013.93 PMC10878732

[18] Consideration of sex differences is necessary to achieve health equity. Nat Rev Nephrol. 2024;20(1):1. 10.1038/s41581-023-00792-z 38036663

[19] Chesnaye NC, et al. Differences in the epidemiology, management and outcomes of kidney disease in men and women. Nat Rev Nephrol. 2024;20(1):7–20. 37985869 10.1038/s41581-023-00784-z

[20] Hart A, et al. OPTN/SRTR 2019 annual data report: kidney. Am J Transpl. 2021;21(Suppl 2):21–137. 10.1111/ajt.16502 33595191

[21] Bloembergen WE, et al. Association of gender and access to cadaveric renal transplantation. Am J Kidney Dis. 1997;30(6):733–8. 9398115 10.1016/s0272-6386(97)90076-7

[22] Segev DL, et al. Age and comorbidities are effect modifiers of gender disparities in renal transplantation. J Am Soc Nephrol. 2009;20(3):621–8. 19129311 10.1681/ASN.2008060591 PMC2653677

[23] Wolfe RA, et al. Differences in access to cadaveric renal transplantation in the united States. Am J Kidney Dis. 2000;36(5):1025–33. 11054361 10.1053/ajkd.2000.19106

[24] Schachtner T, Otto NM, Reinke P. Two decades of the Eurotransplant senior program: the gender gap in mortality impacts patient survival after kidney transplantation. Clin Kidney J. 2020;13(6):1091–100. 33391754 10.1093/ckj/sfz118 PMC7769544

[25] Salter ML, et al. Age and sex disparities in discussions about kidney transplantation in adults undergoing Dialysis. J Am Geriatr Soc. 2014;62(5):843–9. 24801541 10.1111/jgs.12801 PMC4024077

[26] Porrett PM. Biologic mechanisms and clinical consequences of pregnancy alloimmunization. Am J Transpl. 2018;18(5):1059–67. 10.1111/ajt.14673 29369525

[27] Bromberger B, et al. Pregnancy-Induced sensitization promotes sex disparity in living donor kidney transplantation. J Am Soc Nephrol. 2017;28(10):3025–33. 28483798 10.1681/ASN.2016101059 PMC5619956

[28] Klein SL, Flanagan KL. Sex differences in immune responses. Nat Rev Immunol. 2016;16(10):626–38. 27546235 10.1038/nri.2016.90

[29] von Samson-Himmelstjerna FA, et al. The German transplantation registry reveals deficiencies in the listing process for kidney transplantation. Kidney Int Rep. 2023;8(12):2701–8. 38106602 10.1016/j.ekir.2023.09.031 PMC10719593

[30] Dirix M, et al. Timing of the pre-transplant workup for renal transplantation: is there room for improvement? Clin Kidney J. 2022;15(6):1100–8. 35664264 10.1093/ckj/sfac006 PMC9155241

[31] Citarda S, et al. [Access to kidney transplantation’s waiting list: setting up a clinical pathway]. Nephrol Ther. 2016;12(7):525–9. 27771192 10.1016/j.nephro.2016.05.009

[32] Monson RS, et al. Disparities in completion rates of the medical prerenal transplant evaluation by race or ethnicity and gender. Transplantation. 2015;99(1):236–42. 25531896 10.1097/TP.0000000000000271 PMC4274742

[33] Nimmo A, et al. The global impact of COVID-19 on solid organ transplantation: two years into a pandemic. Transplantation. 2022;106(7):1312–29. 35404911 10.1097/TP.0000000000004151 PMC9213067

[34] Putzer G, et al. Solid organ donation and transplantation activity in the Eurotransplant area during the first year of COVID-19. Transplantation. 2022;106(7):1450–4. 35411875 10.1097/TP.0000000000004158 PMC9213062

[35] Nitsch D, et al. Outcomes in patients on home haemodialysis in England and Wales, 1997–2005: a comparative cohort analysis. Nephrol Dial Transpl. 2011;26(5):1670–7. 10.1093/ndt/gfq561 20841489

[36] Mehrotra R, et al. Similar outcomes with Hemodialysis and peritoneal Dialysis in patients with end-stage renal disease. Arch Intern Med. 2011;171(2):110–8. 20876398 10.1001/archinternmed.2010.352

[37] Schold JD, et al. Barriers to evaluation and wait listing for kidney transplantation. Clin J Am Soc Nephrol. 2011;6(7):1760–7. 21597030 10.2215/CJN.08620910

[38] Godara S, Jeswani J. Women donate, men receive: gender disparity among renal donors. Saudi J Kidney Dis Transpl. 2019;30(6):1439–41. 31929292 10.4103/1319-2442.275489

[39] Piccoli GB, et al. What we do and do not know about women and kidney diseases; questions unanswered and answers unquestioned: reflection on world kidney day and international woman’s day. Physiol Int. 2018;105(1):1–18. 29602290 10.1556/2060.105.2018.1.6

[40] Zimmerman D, et al. Gender disparity in living renal transplant donation. Am J Kidney Dis. 2000;36(3):534–40. 10977785 10.1053/ajkd.2000.9794

[41] Rota-Musoll L, et al. An intersectional gender analysis in kidney transplantation: women who donate a kidney. BMC Nephrol. 2021;22(1):59. 33593306 10.1186/s12882-021-02262-9 PMC7885450

[42] Whelan AM, et al. Kidney transplant candidacy evaluation and waitlisting practices in the united States and their association with access to transplantation. Am J Transpl. 2022;22(6):1624–36. 10.1111/ajt.17031 PMC9177783 35289082

[43] Tucker EL, et al. Life and expectations post-kidney transplant: a qualitative analysis of patient responses. BMC Nephrol. 2019;20(1):175. 31096942 10.1186/s12882-019-1368-0 PMC6524208

