# Supplementary material for: Women at disadvantage – sex differences in time between dialysis initiation and kidney transplant evaluation or listing: a single-center experience
Source: BMC Nephrol. 2025 Dec 1;26:691. doi: 10.1186/s12882-025-04663-6 (PMC12696951; doi:10.1186/s12882-025-04663-6)
Supplement: Supplementary file 1 — Supplementary Material 1 [file 12882_2025_4663_MOESM1_ESM.pdf]

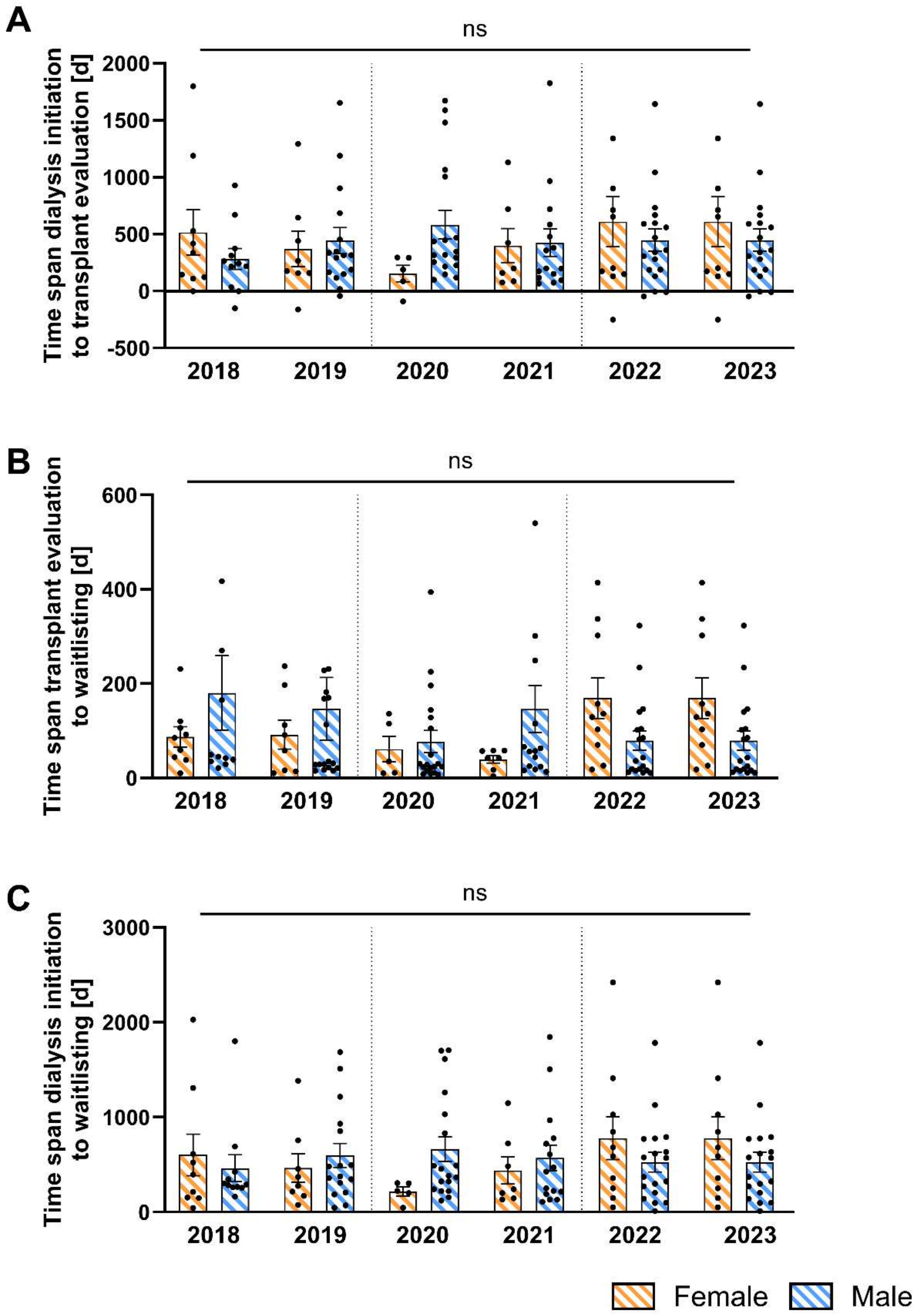

Supplemental Figure 1. Temporal trends from 2018 to 2023. Time intervals between (A) dialysis initiation and transplant evaluation, (B) transplant evaluation and waitlisting, and (C) dialysis initiation and waitlisting stratified by year of transplant evaluation. Statistical analysis was performed using the Kruskal–Wallis test followed by Dunn's post hoc test for multiple comparisons. (ns = not significant)
